# Differential Maintenance of Cortical and Cancellous Bone Strength Following Discontinuation of Bone-Active Agents

**DOI:** 10.1002/jbmr.249

**Published:** 2010-09-13

**Authors:** Mohammad Shahnazari, Wei Yao, Bob Wang, Brian Panganiban, Robert O Ritchie, Yolanda Hagar, Nancy E Lane

**Affiliations:** 1 Department of Medicine, University of California Davis Medical CenterSacramento, CA, USA; 2 Materials Sciences Division, Lawrence Berkeley National LaboratoryBerkeley, CA, USA; 3 Department of Materials Science and Engineering, University of California BerkeleyBerkeley, CA, USA; 4 Division of Biostatistics, University of California DavisDavis, CA, USA

**Keywords:** BONE STRENGTH, TREATMENT WITHDRAWAL, ALENDRONATE, PTH, RALOXIFENE

## Abstract

Osteoporotic patients treated with antiresorptive or anabolic agents experience an increase in bone mass and a reduction in incident fractures. However, the effects of these medications on bone quality and strength after a prolonged discontinuation of treatment are not known. We evaluated these effects in an osteoporotic rat model. Six-month-old ovariectomized (OVX) rats were treated with placebo, alendronate (ALN, 2 µg/kg), parathyroid hormone [PTH(1–34); 20 µg/kg], or raloxifene (RAL, 2 mg/kg) three times a week for 4 months and withdrawn from the treatments for 8 months. Treatment with ALN, PTH, and RAL increased the vertebral trabecular bone volume (BV/TV) by 47%, 53%, and 31%, with corresponding increases in vertebral compression load by 27%, 51%, and 31%, respectively (*p* < .001). The resulting bone strength was similar to that of the sham-OVX control group with ALN and RAL and higher (*p* < .001) with PTH treatment. After 4 months of withdrawal, bone turnover (BFR/BS) remained suppressed in the ALN group versus the OVX controls (*p* < .001). The vertebral strength was higher than in the OVX group only in ALN-treated group (*p* < .05), whereas only the PTH-treated animals showed a higher maximum load in tibial bending versus the OVX controls (*p* < .05). The vertebral BV/TV returned to the OVX group level in both the PTH and RAL groups 4 months after withdrawal but remained 25% higher than the OVX controls up to 8 months after withdrawal of ALN (*p* < .05). Interestingly, cortical bone mineral density increased only with PTH treatment (*p* < .05) but was not different among the experimental groups after withdrawal. At 8 months after treatment withdrawal, none of the treatment groups was different from the OVX control group for cortical or cancellous bone strength. In summary, both ALN and PTH maintained bone strength (maximum load) 4 months after discontinuation of treatment despite changes in bone mass and bone turnover; however, PTH maintained cortical bone strength, whereas ALN maintained cancellous bone strength. Additional studies on the long-term effects on bone strength after discontinuation and with combination of osteoporosis medications are needed to improve our treatment of osteoporosis. © 2011 American Society for Bone and Mineral Research.

## Introduction

A number of drugs offer some protection against postmenopausal bone loss. Alendronate (ALN) and raloxifene (RAL) are antiresorptive agents that attenuate the decline in bone mineral density (BMD) and the risk of vertebral fractures in postmenopausal women.(1,2) Parathyroid hormone (PTH), given intermittently, is an anabolic agent that increases BMD and decreases the risk of vertebral and nonvertebral fractures in postmenopausal osteoporosis.(3) While bisphosphonates such as ALN may accumulate in the skeleton and have continual effects on suppression of bone resorption,(4) the effects of RAL and PTH on bone mass are not maintained long after discontinuation of the drugs, and bone loss resumes within a few months in osteoporotic patients.(5) Several clinical studies have examined the optimal treatment period and changes in areal BMD following discontinuation of these medications, but the effects on bone strength and quality have not been thoroughly investigated. Also, it is not clear whether the anabolic treatment can offer more long-term protection against bone fracture than antiresorptive treatments alone. Cosman and colleagues(6) reported that the PTH-induced bone mass increment in postmenopausal women was maintained for 1 year after discontinuation of PTH and that no clinical fractures occurred if it was continued with estrogen therapy. Lindsay and colleagues(7) reported a sustained vertebral fracture risk reduction up to 18 months after withdrawal of teriparatide in osteoporotic women who had received the drug daily for 18 months. Black and colleagues(5) reported that gains in BMD after 1 year of PTH therapy were maintained if ALN was given, but nearly half the gain was lost if the antiresorptive did not follow. Lane and colleagues(8) showed that PTH-induced increases in bone mass were maintained for 1 year in glucocorticoid-induced osteoporosis when hormone-replacement therapy was continued after PTH withdrawal in postmenopausal women. In most of these studies, osteoporosis drugs were used during the withdrawal time, confounding interpretation of the endpoint variables for durability of the effects related solely to the original medication. As for preclinical studies, very limited data are available on the recovery of bone turnover after withdrawal of bisphosphonate therapy(9) and changes in areal BMD following discontinuation of PTH treatment in rats.(10) Using an osteoporotic rat model, we report a series of bone structural and material properties related to changes in bone turnover and bone mass during withdrawal of antiresorptive and anabolic agents commonly used for treatment of osteoporosis. During treatment, the increases in bone mass in trabecular sites were similar with all drugs, whereas after discontinuation of the treatments, there were treatment-specific differences in cortical versus trabecular bone turnover and mechanical properties.

## Materials and Methods

### Animals and experimental procedures

**Fig. 1:**
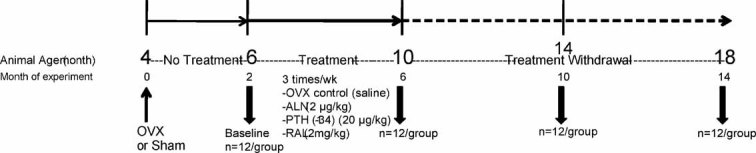
Study design. Treatment started 2 months after OVX surgery in female Sprague-Dawley rats. Animals were treated three times per week for 4 months with either saline (sham and OVX controls), ALN (2 µg/kg), hPTH(1–34) (20 µg/kg), or RAL (2 mg/kg). Twelve animals per treatment group were killed at baseline, after treatment, and at 4 and 8 months after discontinuation of treatment.

Four-month-old female ovariectomized (OVX) or sham-operated Sprague-Dawley rats were purchased from Harlan Industries (Livermore, CA, USA). Rats were maintained on commercial rodent chow (Rodent Diet, Teklad, Madison, WI, USA) with 0.95% calcium and 0.67% phosphorus in a room with 21°C temperature and a 12-hour light/dark cycle. All study animals were pair fed during the entire experiment, approximately 18 g of rodent chow a day, to prevent excessive postovariectomy weight gain. The study protocol was approved by University of California Davis Institutional Animal Care and Use Committee. The OVX rats were left untreated for 2 months to develop osteopenia. Figure 1 summarizes the study timeline and the experimental groups. At 6 months of age, pretreatment measurements were obtained from sham-operated and OVX groups (*n* = 12/group). The remaining OVX animals were randomized by body weight into four groups (*n* = 36/group): OVX control [treated with saline three times per week by subcutaneous (s.c.) injection], ALN (2 µg/kg/dose three times per week by s.c. injection), PTH(1–34) (20 µg/kg/dose three times per week by s.c. injection), and RAL (2 mg/kg/dose three times per week by oral gavage). Treatments were given for 4 months, after which 12 rats from each group were killed, and the remaining animals were continued for an additional 4 or 8 months of withdrawal with no more active treatments, when 12 rats per treatment group were killed at each time point (Fig. 1).

Urine samples were collected from all experimental groups for the deoxypyridinoline (DPD) assay of bone resorption at baseline and during treatment and withdrawal periods. In vivo micro–computed tomographic (µCT) scans of the proximal tibia were obtained during the treatment and withdrawal periods to monitor the changes in bone mass and microarchitecture. Ex vivo µCT measurements were obtained from the fifth lumbar vertebra (L_5_) and femoral mid-diaphysis for bone mass and architectural evaluations, as well as cortical mineral density. Histomorphometric measurements of surface-based bone turnover were obtained from the proximal tibial metaphyses and the midshaft of the tibia from all animals by labeling the skeleton with calcein (10 mg/kg s.c.; Sigma-Aldrich, St Louis, MO, USA) 10 and 3 days before killing at each time point. Bone mechanical properties were examined by compression test of the sixth lumbar vertebra (L_6_) and bending test of the left tibia. The compression test of the vertebral bodies also was simulated using finite-element analyses of mineral and architectural data to estimate bone stiffness and the apparent modulus.(11) Details of each measurement are presented below.

### µCT measurements of bone architecture and mineral density of bone tissue

In vivo scans of the proximal tibial metaphysis (PTM) were obtained after 4 months of treatment and at 2, 4, and 8 months during treatment withdrawal using µCT (*viva* CT40, Scanco Medical, Bassersdorf, Switzerland). This allowed monitoring of the changes in BV/TV and bone microarchitecture after the discontinuation of treatments. Rats were anesthetized with isoflurane during the entire scan, which lasted about 20 minutes. The scans were obtained at a high resolution of 10.5 µm from the tibial metaphysial region 1.5 mm below the growth plate and extending for 2.2 mm. A 2D TIF image with reference lines was saved from each scan to ensure that the same region of scan was evaluated during serial imaging. Ex vivo µCT scans were obtained from the fifth lumbar vertebral bodies (L_5_) and femoral midshafts for all the animals studied. A 2.2-mm-long central cross-sectional region of each vertebra was scanned at an energy level of 70 kVp and intensity of 85 µA with an isotropic resolution of 10.5 µm in all three spatial dimensions. Bone volume/total volume (BV/TV), trabecular thickness (Tb.Th), trabecular number (Tb.N), connectivity density, and separation (Tb.Sp) are reported for each vertebral site. In addition, the femoral midshaft was scanned (95 slices with 10.5-µm resolution and 325-ms integration time) to evaluate changes in cortical bone volume, architecture, and mineral density. The scan region was selected using reference lines positioned at the top of femoral head and at the base of medial condyle.

### Deoxypyridinoline assay

Urinary DPD and creatinine (Cr) were measured using an EIA kit (Quidel, San Diego, CA, USA) at baseline, 1 month after treatments began and were withdrawn, and bimonthly thereafter throughout the study. The assay provided a measure of excretion of DPD cross-links as an indicator of bone resorption. The urine samples were collected by placing rats in individual metabolic cages and were diluted 50 times with deionized water. The DPD and Cr concentrations of samples were determined in duplicate using a microplate reader (SpectraMax M2, Molecular Devices, Sunnyvale, CA, USA) and according to the manufacturer's recommendations. The DPD results were corrected for variations in urine concentration by dividing the values by the Cr value. Samples with high concentrations or coefficients of variation over 10% were further diluted or repeated for measurement to fit into the four-parameter calibration curve used to calculate the concentrations, as reported previously from this laboratory.(12)

### Bone histomorphometry measurements

Dynamic bone histomorphometric measures were obtained from proximal (PTM) and midshaft tibias (TX) of each animal. Bone samples were fixed, dehydrated, and embedded undecalcified in methyl methacrylate. Metaphyseal sections of 4 and 8 µm thick were cut using a microtome (Leica RM 2265, Leica Microsystems, Nussloch GmbH, Germany). Mid-diaphyseal cross sections 40 µm thick were cut using a diamond wire saw (Well 3241, Norcross, GA, USA). The 4-µm sections were stained with tetrachrome and mounted with Permount. The 8-µm PTM and 40-µm TX sections were left unstained for fluorescent microscopy using image-analysis software (Bioquant Image Analysis Corporation, Nashville, TN, USA). Bone areas and perimeters were measured at a magnification of ×25 and indices of bone formation and cell surfaces at ×250. Bone measurements included percent of trabecular bone surface covered by osteoclasts (Oc.S/BS) and single- and double-labeled perimeters and interlabeled width, from which mineralizing surface (MS/BS), mineral apposition rate (MAR), and bone-formation rate (BFR/BS) were calculated according to the ASBMR guidelines for bone histomorphometry.(13)

### Biomechanical testing

Mechanical properties of bone were determined by compression of lumbar vertebral body (L_6_) and bending of tibia. The vertebral endplates were removed using a wafer saw and polished to flat surfaces. The height of the vertebral body and the average caudal and cranial diameters were measured using a caliper for calculation of cross-sectional area and estimation of material properties. The bone was loaded using an electroservohydraulic materials testing system (Model 831, MTS, Eden Prairie, MN, USA) at a displacement rate of 0.01 mm/s. Maximum load, yield stress, maximum stress, and the elastic modulus were obtained from the compression tests of vertebral bodies. A similar displacement rate was used to measure tibial strength in a three-point bending test after cutting off the proximal and distal ends. The bone specimens were loaded such that the posterior surface was in tension and the anterior surface in compression. Following bending, the broken halves of bones were examined for the fracture surface in an environmental scanning electron microscope (Hitachi S-4300SE/N ESEM, Hitachi America, Pleasanton, CA, USA). The cross-sectional area and the second moment of inertia were calculated from the scanning electron microscopic (SEM) image taken using ImageJ software (NIH: http://rsb.info.nih.gov/ij) from fracture surfaces. The yield stress was determined at 0.2% plastic strain and the maximum stress at peak load.(14) Elastic modulus values in bending and compression were calculated from the slope of the linear region of the stress-strain curve.

To analyze the biomechanical properties of the vertebral body with relative contribution of the cortical shell and the trabecular core, a µCT-based finite-element model was used for each rat vertebra with a 10.5-µm voxel size. The model simulated uniaxial vertebral compression loading with the cranial and caudal ends fixed in between two loading planes. The cortical and trabecular bone were segmented by manually tracing the endosteal surface of the cortex for every 15 slices from each scan of 2.2 mm obtained from the central vertebral body, where the bone mass and architectural parameters were evaluated. The µCT images then were incorporated into the model as described by Ladd and colleagues.(15) The 3D image voxels were converted to elements, and each element segmented as bone was assigned a Young's modulus of 18 GPa and a Poisson's ratio of 0.3, as reported previously.(16) Details of the numerical method have been published elsewhere.(17,18) The boundary conditions that defined the load platen-specimen interface were assumed in the model to be frictionless. Vertebral stiffness, apparent modulus, trabecular tissue strength, and changes in the load-carrying capacity of the vertebral trabecular bone with treatments were calculated from finite-element analyses, as reported previously by this group.(12)

### Statistical analysis

Initial summary statistics (mean and SE) were calculated for the outcome, bone strength, which was measured with the vertebral maximum load variable, and for five bone structure covariates of interest: vertebral trabecular BV/TV, vertebral Tb.Th, vertebral Tb.N, vertebral cortical bone volume (BV), and vertebral cross-sectional area.

We then investigated three possible mechanistic hypotheses: (1) Bone structure is affected by treatment and, in turn, affects vertebral maximum load, (2) bone structure and vertebral maximum load are both affected by treatment, and (3) treatment and bone structure both affect vertebral maximum load. To determine what role each bone structure covariate may have in affecting vertebral maximum load, we created two types of models: (1) linear regression models that examine the effects of OVX, treatment, and treatment withdrawal on each bone structure measurement (this was done separately for vertebral trabecular BV/TV, vertebral cortical BV and Ct.Th, vertebral Tb.Th, vertebral Tb.N, and vertebral cross sectional area) and (2) linear regression models that examined the changes in treatment effects (both after treatment and during treatment withdrawal) on vertebral maximum load when bone structure covariates are included individually. This was done separately for vertebral trabecular BV/TV, vertebral Tb.Th, vertebral Tb.N vertebral cortical BV and Ct.Th, and vertebral cross-sectional area. To get initial information on µCT measurements of vertebral trabecular and femoral cortical bone and urinary DPD, ANOVAs were created at each time point. Most calculations were performed in R 2.0 (http://www.r-project.org), with the ANOVAs created in Minitab (Minitab, Inc., State College, PA, USA).

## Results

The study medications generally were well tolerated over the 14- month study period; however, 8 of 215 rats (approximately 4%) were removed from the study owing to development of tumors of different types. Administration of the study medications did not affect the weights of the rats.

### µCT measurements of vertebral trabecular and femoral cortical bone

**Fig. 2:**
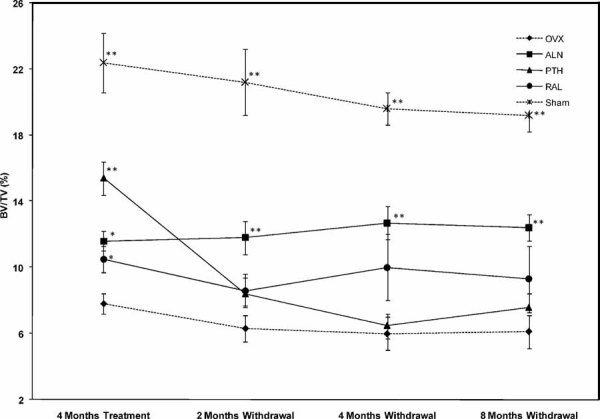
In vivo µCT scans of proximal tibia metaphysis in rats after 4 months of treatment with ALN, hPTH(1–34), and RAL and at 2, 4, and 8 months following treatment withdrawal. Double and single asterisks indicate *p* < .001 and *p* < .05, respectively, for comparison with OVX control group at each time point.

The data obtained using µCT from in vivo imaging of the proximal tibia 2 months after discontinuation of treatment indicated no difference between the PTH or RAL groups and OVX controls in BV/TV (Fig. 2), Tb.N, and trabecular connectivity; however, the ALN group had higher values than the OVX control group for all these parameters (*p* < .001), which remained higher until 8 months following treatment withdrawal. The ex vivo vertebral bone mass and architectural results are presented in Table 1. ALN, PTH, and RAL increased the vertebral trabecular BV/TV of OVX animals by 47%, 53%, and 31%, respectively (*p* < .001), which was comparable with sham only with PTH treatment. Tb.Th was similar to sham with ALN and RAL and higher than sham with PTH treatment (*p* < .001), whereas Tb.N was restored to the sham level only with ALN treatment. µCT scans of the midshaft of the femur showed an increase in Ct.Th with all active treatments (*p* < .001), but cortical BV increases were significant only in the ALN (*p* < .001) and PTH (*p* < .05) groups. Of the active treatments, only PTH increased the cortical mineral density compared with untreated OVX animals (1138 versus 1125 mg HA/cm^3^, *p* < .05; Table 1.

**Table 1:** Vertebral and Femoral Bone Mass and Microarchitectural Parameters in OVX Rats Following Administration and Withdrawal of Treatment With ALN, hPTH(1–34), and RAL (Mean ± SE)

	4 mo Treatment (Age: 10 mo)					4 mo withdrawal (Age: 14 mo)					8 mo withdrawal (Age: 18 mo)				
			
	Sham	OVX	OVX + ALN	OVX + PTH	OVX + RAL	Sham	OVX	OVX + ALN	OVX + PTH	OVX + RAL	Sham	OVX	OVX + ALN	OVX + PTH	OVX + RAL
Vertebral Trabecular Bone (ex vivo)															
Bone Volume/Total Volume (%)	31.8 ± 1.0**	19.2 ± 1.6	28.2 ± 1.3**	29.4 ± 1.2**	25.2 ± 1.5*	34.6 ± 1.1**	18.6 ± 1.4	22.8 ± 1.3*	19.0 ± 1.7	21.0 ± 2.2	33.3 ± 1.4**	18.9 ± 1.5	23.3 ± 1.0*	21.4 ± 1.2	18.8 ± 1.0
Trabecular Thickness (µm)	81.5 ± 1.6**	75.3 ± 1.7	81.6 ± 1.7*	95.8 ± 1.8**	83.7 ± 2.0**	89.2 ± 2.0**	80.7 ± 1.4	79.9 ± 1.3	76.0 ± 1.8	80.4 ± 1.5	88.1 ± 1.9**	77.6 ± 2	80.3 ± 1.4	80.2 ± 1.2	80.0 ± 1.6
Trabecular Number (1/mm)	3.9 ± 0.1**	2.9 ± 0.1	3.5 ± 0.1**	3.0 ± 0.1	3.1 ± 0.1	3.8 ± 0.1**	2.8 ± 0.1	3.2 ± 0.1*	2.7 ± 0.1	2.9 ± 0.1	3.8 ± 0.1**	2.8 ± 0.1	3.1 ± 0.1*	2.8 ± 0.1	2.7 ± 0.1
Connectivity Density (1/mm^3^)	69.4 ± 3.3	55.3 ± 6.9	59.9 ± 5.0	37.0 ± 1.8*	44.4 ± 2.8	51.0 ± 2.7**	38.3 ± 2.0	49.4 ± 2.9**	42.6 ± 2.5	44.2 ± 3.5	44.0 ± 2.6	39.7 ± 3.3	53.2 ± 2.7**	45.6 ± 2.2	37.7 ± 2.7
Trabecular Separation (µm)	254 ± 9**	352 ± 17	276 ± 12**	323 ± 11	312 ± 13	256 ± 6**	350 ± 17	302 ± 8*	384 ± 20	353 ± 18	261 ± 7**	344 ± 19	308 ± 7*	362 ± 13	374 ± 17
Mineral Density of Bone Tissue (mgHA/cm^3^)	1119 ± 9*	1100 ± 8	1092 ± 18	1107 ± 14	1105 ± 9	1115 ± 14	1105 ± 6	1106 ± 13	1099 ± 12	1099 ± 10	1135 ± 11	1118 ± 13	1123 ± 10	1116 ± 11	1120± 11
Femoral Cortical Bone															
Cortical Thickness (mm)	0.66 ± 0.01	0.63 ± 0.01	0.68 ± 0.01**	0.68 ± 0.01**	0.68 ± 0.01*	0.67 ± 0.01	0.64 ± 0.01	0.69 ± 0.01**	0.64 ± 0.01	0.65 ± 0.01	0.67 ± 0.01*	0.64 ± 0.01	0.70 ± 0.01**	0.67 ± 0.01	0.64 ± 0.01
Bone Volume (mm^3^)	5.53 ± 0.09*	5.81 ± 0.07	6.28 ± 0.1**	6.13 ± 0.1*	6.0 ± 0.1	5.80 ± 0.3	5.95 ± 0.3	6.25 ± 0.3*	6.07 ± 0.3	6.04 ± 0.3	5.76 ± 0.04	5.87 ± 0.1	6.51 ± 0.06**	6.23 ± 0.1*	6.15 ± 0.2
Mineral Density of Bone Tissue (mgHA/cm^3^)	1133 ± 6	1125 ± 5	1127 ± 5	1138 ± 4*	1129 ± 3	1120 ± 4	1123 ± 3	1121 ± 4	1123 ± 2	1126 ± 4	1135 ± 2	1129 ± 6	1143 ± 3*	1125 ± 6	1125 ± 4

* Different from OVX (*p* < .05) in the same age group.

** Different from OVX (*p* < .001) in the same age group.

**Fig. 3:**
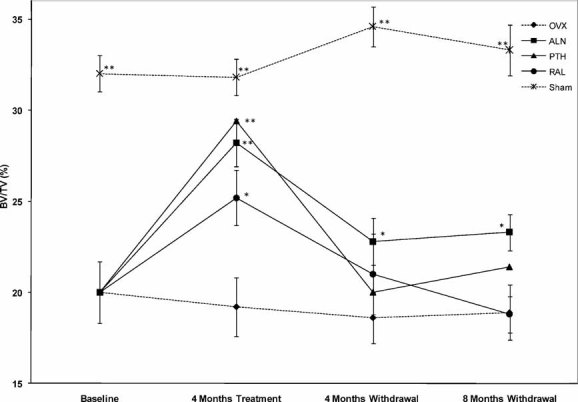
Timeline effect of ALN, hPTH(1–34), and RAL on vertebral trabecular bone volume (BV/TV) of OVX rats and changes at 4 and 8 months after withdrawal of the treatments. All three drug treatments increased BV/TV of OVX animals. PTH and RAL withdrawal resulted in a fall of BV/TV to OVX control levels, whereas animals withdrawn from ALN treatment maintained a 20% higher BV/TV than OVX animals for 8 months. Double and single asterisks indicate *p* < .001 and *p* < .05, respectively, for comparison with OVX control group at each time point.

Animals withdrawn from PTH and RAL treatment appeared no different from their OVX control in vertebral BV/TV and architectural parameters. However, animals withdrawn from ALN had at least 20% higher BV/TV than their OVX controls (*p* < .05) that was maintained up to 8 months of withdrawal. They also showed higher Tb.N (*p* < .05) and connectivity density (*p* < .001) and lower Tb.Sp (*p* < .05) than the OVX control group. Similarly, the femoral cortical BV and Ct.Th were higher than in the OVX animals only in ALN-withdrawn animals and up to 8 months (*p* < .001). The overall effect of administration and withdrawal of treatments on vertebral trabecular BV/TV is shown in Figure 3.

### Urinary deoxypyridinoline

**Fig. 4:**
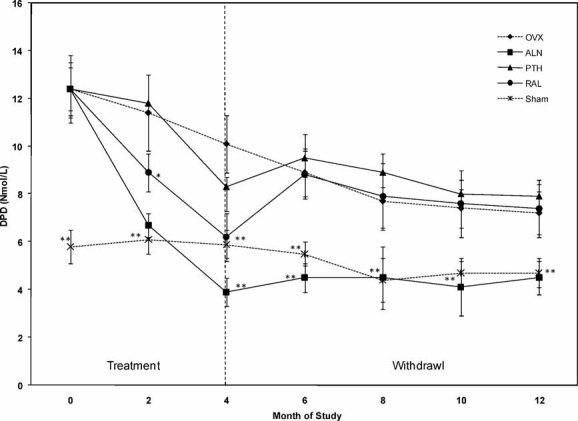
Urinary DPD cross-links of collagen adjusted for urinary creatinine level in rats from different experimental groups during treatment and treatment withdrawal periods. Double and single asterisks indicate *p* < .001 and *p* < .05, respectively, for comparison with OVX control group at each time point.

Osteoclast activity was determined by measurement of the bone-related degradation product of DPD cross-links in urine. As expected, the OVX control rats showed an increase in DPD (*p* < .001), which was maintained higher than in the sham group throughout the experiment (Fig. 4). Treatment with ALN decreased the levels of urinary DPD to those seen in the sham group and to lower than sham levels after 4 months of treatment (*p* < .05). The urinary levels of DPD in ALN-treated rats remained at sham control levels during the 8-month withdrawal period. Raloxifene also reduced the OVX-induced rise in urinary DPD soon after initiation of the treatment such that after 4 months of treatment, the DPD levels were as low as those observed in the sham group. However, 2 months after discontinuation of RAL, DPD levels increased to OVX control group levels and remained high for the rest of the experiment. Treatment with PTH did not affect the levels of urinary DPD compared with the OVX control group, although there was a tendency to higher values after discontinuation of treatment.

### Bone histomorphometry

Histomorphometric results of tibial cancellous and cortical bone are summarized in Table 2. Compared with the OVX control group, treatment with ALN, PTH, and RAL increased trabecular BV/TV by 71%, 90%, and 43%, respectively, with only the PTH mean value being similar to that of sham group. Histomorphometry also revealed differences in the cellular response to drugs. OVX animals had a 100% increase in Oc.S./BS versus the sham group. Treatment with PTH further increased Oc.S./BS, whereas ALN and RAL treatments reduced Oc.S./BS in OVX rats to sham levels (*p* < .01).

**Table 2:** Histomorphometric Parameters of Tibial Metaphyses and Mid-Diaphyses in OVX Rats Following Administration and Withdrawal of Treatment With ALN, hPTH(1–34), and RAL (Mean ± SE)

	4 mo Treatment (Age: 10 mo)					4 mo withdrawal (Age: 14 mo)					8 mo withdrawal (Age: 18 mo)				
			
	Sham	OVX	OVX + ALN	OVX + PTH	OVX + RAL	Sham	OVX	OVX + ALN	OVX + PTH	OVX + RAL	Sham	OVX	OVX + ALN	OVX + PTH	OVX + RAL
Tibial Metaphyses															
BV/TV (%)	19.6 ± 1.2**	8.6 ± 1	14.7 ± 1.3**	16.4 ± 1.6**	12.3 ± 1.0*	19.7 ± 0.8**	6.8 ± 0.5	11.2 ± 0.7**	7.6 ± 0.5	7.7 ± 0.7	16.7 ± 1.3**	4.6 ± 0.7	11.0 ± 1.3**	7.1 ± 1.1	6.6 ± 1.0
Oc.S/BS (%)	3.2 ± 0.3**	6.5 ± 0.3	3.0 ± 0.4**	7.1 ± 0.4	3.2 ± 0.3**	2.9 ± 0.4**	6.2 ± 0.4	3.1 ± 0.2**	6.8 ± 0.4	4.9 ± 0.4	3.5 ± 0.3**	5.5 ± 0.5	3.7 ± 0.2*	5.7 ± 0.4	6.3 ± 0.6
MS/BS (%)	23.6 ± 1.3*	27.5 ± 1.2	11.3 ± 0.8**	34.9 ± 1.5**	21.0 ± 1.1**	24.8 ± 1.5*	30.8 ± 1.9	21.2 ± 1.3*	35.6 ± 2*	36.1 ± 3.0*	19.8 ± 2.4*	24.3 ± 0.9	17.6 ± 2.1*	23.1 ± 2.7	27.6 ± 2.7
MAR (µm/d)	1.35 ± 0.05*	1.51 ± 0.06	0.83 ± 0.03**	1.58 ± 0.06	1.32 ± 0.06*	1.11 ± 0.07	1.25 ± 0.06	1.08 ± 0.06	1.76 ± 0.07**	1.68 ± 0.07**	0.62 ± 0.1	0.76 ± 0.06	0.50 ± 0.04*	0.81 ± 0.1	0.79 ± 0.09
BFR/BS (µm^3^/µm^2^/d)	0.32 ± 0.02*	0.41 ± 0.02	0.10± 0.01**	0.55 ± 0.04**	0.27 ± 0.02**	0.27 ± 0.03*	0.39 ± 0.04	0.23± 0.03**	0.62 ± 0.05**	0.60 ± 0.06**	0.12 ± 0.01*	0.18 ± 0.01	0.09± 0.01**	0.19 ± 0.01	0.22 ± 0.01
Tibial Endocortical Mid-diaphyses															
BFR/BS (µm^3^/µm^2^/d)	0.02 ± 0.004**	0.2 ± 0.05	0.05 ± 0.02*	0.05 ± 0.01**	0.09 ± 0.03*	0.06 ± 0.008	0.09 ± 0.01	0.11 ± 0.01	0.13 ± 0.01*	0.18 ± 0.05*	0.04 ± 0.008	0.04 ± 0.007	0.03 ± 0.004	0.03 ± 0.02	0.07 ± 0.028

BV/TV = bone volume/total volume; Oc.S./BS = osteoclast surface/bone surface; MS/BS = mineralizing surface/bone surface; MAR = mineral apposition rate; BFR/BS = bone-formation rate/bone surface.

* Different from OVX (*p* < .05) of same age group.

** Different from OVX (*p* < .001) of same age gtoup.

The endocortical BFR/BS was 10-fold higher in OVX versus sham groups and suppressed by 55% to 75% with antiresorptive treatments. Following withdrawal of the treatments, only rats in the ALN group had a higher BV/TV versus the OVX group (*p* < .001), consistent with their lower Oc.S/BS. Rats withdrawn from RAL and PTH treatments showed higher rates of MS/BS (*p* < .05), MAR (*p* < .001), and BFR/BS (*p* < .001) for 4 months, but after 8 months of withdrawal, the values were not different from those of the OVX control group. Surface-based bone formation, as measured by BFR/BS, was suppressed with ALN and RAL by 74% and 35%, respectively (*p* < .001), and increased with PTH by 35%. The increase in BFR/BS with PTH was mainly due to an increase in bone mineralizing surface.

The endocortical BFR/BS after 4 months of withdrawal was higher than in the OVX control group in the PTH- and RAL-treated groups only (*p* < .05); however, it appeared unchanged across all experimental groups after 8 months of withdrawal. The periosteal bone formation, except for a very few animals in the OVX and PTH groups at 4 months of withdrawal, was essentially absent in all the treatment and withdrawal groups.

### Mechanical testing for whole and localized bone strength

Table 3 summarizes the bone mechanical properties obtained from lumbar compression and tibial bending tests. Compared with the sham group, vertebral bone in OVX control animals showed decreases in maximum load (*p* < .05), yield stress (*p* < .001), maximum stress (*p* < .001), and elastic modulus (*p* < .05). Increases in vertebral maximum load were observed following treatment with ALN (27%, *p* < .001), PTH (51%, *p* < .001), and RAL (31%, *p* < .05), with the PTH group being even higher than the sham control group (*p* < .001). Adjusting the structural strength by vertebral geometry, the increases in maximum stress were 16%, 37%, and 21% versus the OVX control group (*p* < .001 for ALN and PTH and *p* < .05 for RAL), respectively. For the tibia, only the maximum stress decreased with ovariectomy (*p* < .05), although there were nonsignificant decreases in yield stress and elastic modulus. Compared with the vertebral improvements in strength, the increases in tibial bending strength with the treatments were of smaller magnitude, 6% to 11%, but statistically significant (*p* < .05). None of the parameters of maximum stress, yield stress, or bending modulus of tibia showed any improvement with the drug treatments.

**Table 3:** Mechanical Properties of Vertebrae and Tibias in OVX Rats Treated for 4 Months With ALN, hPTH(1–34), and RAL and Followed for 4 and 8 Months After Discontinuation of the Drugs (Mean ± SE)

	4 mo Treatment (Age:10 mo)					4 mo Withdrawal (Age: 14 mo)					8 mo Withdrawal (Age: 18 mo)				
			
	Sham	OVX	OVX+ ALN	OVX+ PTH	OVX+ RAL	Sham	OVX	OVX+ ALN	OVX+ PTH	OVX+ RAL	Sham	OVX	OVX+ ALN	OVX+ PTH	OVX+ RAL
Body Weight (g)	270.8 ± 2.9*	320.0 ± 5.8	315.5 ± 6.7	318.9 ± 5.1	308.7 ± 7.1	283.6 ± 3.3*	330.0 ± 7.7	338.0 ± 4.1	346.7 ± 6.0	339.7 ± 6.4	296.0 ± 4.9*	330.0 ± 5.8	336.7 ± 8.2	333.9 ± 7.7	346.2 ± 6.1
Vertebral Compression Test															
Maximum Load (N)	244.5 ± 10.2*	202.6 ± 10.7	258.5 ± 13.2**	305.1 ± 14.2**	266.0 ± 19.2*	260.5 ± 20.9*	222.1 ± 9.0	257.7 ± 16.1*	229.6 ± 16.6	225.8 ± 18.0	264.4 ± 14.1**	207.6 ± 13.2	217.5 ± 8.3	209.3 ± 14.1	196.6 ± 11.4
Yield Stress (MPa)	18.8 ± 1.0**	13.6 ± 0.8	12.5 ± 1.4	17.5 ± 1.8*	15.8 ± 1.3	15.4 ± 1.2*	12.1 ± 0.8	12.1 ± 1.1	11.7 ± 1.2	11.1 ± 1.1	17.4 ± 1.3*	12.8 ± 0.9	12.3 ± 0.6	12.1 ± 0.8	9.6 ± 0.6*
Maximum Stess (MPa)	21.2 ± 0.8**	15.7 ± 0.6	18.2 ± 0.6**	21.6 ± 1.4**	19.0 ± 1.4*	18.2 ± 1.3*	14.8 ± 0.8	15.5 ± 0.8	13.5 ± 1.0	14.4 ± 1.0	21.1 ± 1.1**	14.7 ± 0.9	15.3 ± 0.6	14.7 ± 0.9	14.1 ± 1.0
Modulus (GPa)	0.58 ± 0.06*	0.39 ± 0.04	0.44 ± 0.05	0.41 ± 0.06	0.51 ± 0.07	0.47 ± 0.06	0.37 ± 0.05	0.39 ± 0.05	0.32 ± 0.05	0.29 ± 0.04	0.54 ± 0.1	0.4 ± 0.05	0.55 ± 0.06	0.36 ± 0.05	0.4 ± 0.05
Tibial Bending Test															
Maximum Load (N)	97.6 ± 1.5	100.7 ± 2.0	105.7 ± 2.8	111.0 ± 2.9**	111.7 ± 3.2**	98.8 ± 2.1	102.5 ± 3.5	103.6 ± 2.5	110.7 ± 1.5*	104.8 ± 2.7	93.0 ± 2.5	100.0 ± 2.6	103.5 ± 3.3	107.0 ± 3.5	102.4 ± 4.4
Maximum Stress (MPa)	172.4 ± 11.2*	143.2 ± 13.9	144.5 ± 13.4	144.8 ± 10.5	148.9 ± 13.6	135.8 ± 7.8	143.4 ± 10.2	145.7 ± 9.4	150.5 ± 9.3	129.8 ± 10.0	151.4 ± 7.8	145.1 ± 7.8	142.3 ± 4.8	147.3 ± 6.2	141.9 ± 3.2
Yield Stress (MPa)	138.2 ± 13.1	116.9 ± 13.4	118.0 ± 12.2	111.9 ± 8.3	111.6 ± 11.1	104.1 ± 6.7	118.7 ± 12.6	106.6 ± 8.9	119.6 ± 11.6	95.3 ± 6.8	124.9 ± 7.7	119.9 ± 8.6	117.4 ± 4.3	116.9 ± 8.6	119.1 ± 5.3
Modulus (GPa)	8.62 ± 0.76	6.66 ± 0.81	6.34 ± 0.71	6.40 ± 0.80	6.15 ± 0.98	6.58 ± 0.46	7.06 ± 0.74	7.10 ± 0.62	6.09 ± 0.67	5.48 ± 0.44	6.75 ± 0.65	6.32 ± 0.45	5.44 ± 0.61	5.98 ± 0.37	4.87 ± 0.25*
Vertebral Compression Estimates by FEA															
Stiffness (N/mm) (×10,000)	2.64 ± 0.06**	2.02 ± 0.10	2.85 ± 0.13**	2.92 ± 0.10**	2.76 ± 0.16**	2.92 ± 0.14*	2.20 ± 0.18	3.10 ± 0.16*	2.95 ± 0.11*	2.80 ± 0.20*	2.82 ± 0.12*	2.18 ± 0.13	2.95 ± 0.11**	2.56 ± 0.10	2.40 ± 0.12
Apparent Modulus (GPa)	5.98 ± 0.14**	4.48 ± 0.18	5.35 ± 0.19**	5.83 ± 0.13**	5.27 ± 0.17**	5.94 ± 0.20**	4.74 ± 0.19	5.64 ± 0.20*	4.92 ± 0.19	5.21 ± 0.25	6.04 ± 0.20**	4.76 ± 0.19	5.55 ± 0.14**	4.89 ± 0.13	4.70 ± 0.14
Average Trab. Tissue Stress (MPa)	117.8 ± 1.2**	104.2 ± 1.8	116.1 ± 1.2**	120.2 ± 1.5**	116.2 ± 1.1**	118.0 ± 1.4**	108.0 ± 1.7	112.6 ± 2.0	101.0 ± 8.6	108.7 ± 2.8	116.7 ± 1.4**	107.0 ± 3.1	110.5 ± 1.5	109.9 ± 1.8	105.5 ± 2.1
Load Carried by Tb. Bone (%)	25.1 ± 1.9*	18.4 ± 1.8	21.0 ± 2.0	21.8 ± 1.8	21.6 ± 1.9	25.5 ± 1.7*	19.5 ± 1.7	18.1 ± 1.4	16.6 ± 1.5	17.5 ± 1.8	27.5 ± 1.5**	18.3 ± 1.1	21.9 ± 1.1*	20.6 ± 1.0	19.2 ± 1.7
Load Carried by Ct. Bone (%)	74.9 ± 1.9*	81.6 ± 1.8	78.9 ± 2.0	79.3 ± 1.6	78.3 ± 1.9	74.5 ± 1.7*	80.5 ± 1.7	81.9 ± 1.4	83.4 ± 1.5	82.5 ± 1.8	72.5 ± 1.5**	81.7 ± 1.1	78.1 ± 1.1*	79.4 ± 1.0	80.8 ± 1.7

* Different from OVX (*p* < .05) in the same age group.

** Different from OVX (*p* < .001) in the same age group.

Four months after withdrawal of the treatments, vertebral strength (maximum load) was still higher than in the OVX control group (*p* < .05) and comparable with the sham group mean value only in the ALN group. The vertebral mechanical improvements resulting from PTH and RAL treatments were no longer detectable at this time point of the experiment. Interestingly, the tibial strength in PTH-treated rats still was higher than in the OVX control group after 4 months of withdrawal (*p* < .05), an effect that was not observed in the ALN or RAL groups. All the active treatments appeared to have lost their mechanical effects 8 months after discontinuation regardless of the skeletal site.

Simulation of the in vitro vertebral compression test using µCT data and finite-element analyses showed increases in vertebral bone stiffness, apparent modulus, and average trabecular tissue stress with all treatments compared with the OVX control group (*p* < .001) and were similar in trend to those observed with bone mass and architectural parameters. The estimated vertebral stiffness and apparent modulus were maintained up to 8 months following withdrawal of ALN (*p* < .001), whereas with PTH and RAL withdrawal, the values returned to the OVX control levels, supporting the in vitro mechanical test results.

### Treatment effect on bone strength with the addition of bone structure covariates

**Fig. 5:**
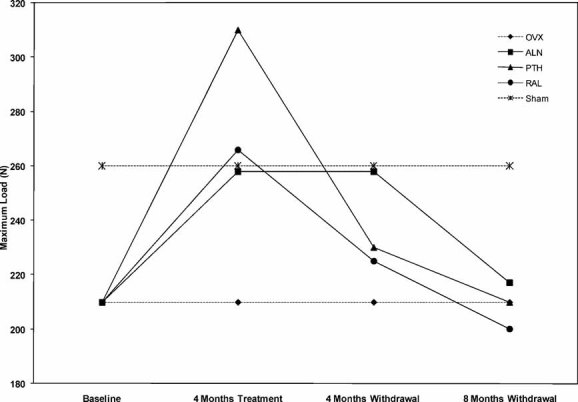
Trajectories of vertebral maximum load with time. Withdrawal of ALN was not followed by a decline in vertebral strength. Withdrawal of PTH or RAL led to loss of vertebral strength by 4 months after treatment cessation but without significant additional decline over the next 4 months.

When no bone structure covariates were included in the prediction of vertebral maximum load (the initial model), after 4 months of treatment, ALN-, PTH-, and RAL-treated rats were 95%, 256%, and 146% higher than untreated OVX rats (*p* = .01, p = .06, and *p* = .01). After 4 months of treatment withdrawal, PTH and RAL rats experienced a 78% and 72% decline in vertebral maximum load measures, meaning that average measures were 20% and 32% lower than in untreated OVX animals. ALN rats did not experience any significant decline in vertebral maximum load after treatment stopped, and none of the treatment groups experienced significant decline between 4 and 8 months of withdrawal (Fig. 5).

All the bone structure covariates, when included individually as predictors in the initial model, were significantly correlated with baseline measures of vertebral maximum load. Baseline maximal load was 96% greater for every 1/10 of a unit increase in vertebral trabecular BV/TV units (*p* < .01), 32% greater for every unit increase in vertebral cortical BV (*p* = .03), 25% greater for every 1/100 of a point increase in vertebral Tb.Th (*p* < .01), 62% greater for every unit increase in vertebral Tb.N. (*p* < .01), and 5% greater for every unit increase in vertebral cross sectional area (*p* < .01).

When added individually as predictors of vertebral maximum load, some of the bone structure covariates reduced the increase in vertebral maximum load measures accounted for by PTH and RAL treatment. When vertebral trabecular BV/TV was included as an explanatory variable, the difference between untreated OVX rats and treated rats at 4 months of treatment was no longer statistically significant (*p* = .67, *p* = .09, and *p* = .13), and there also were no significant decreases for these groups after treatment withdrawal. When vertebral cortical BV, vertebral Tb.Th, or vertebral cross-sectional area was added to the initial model as a predictor, the differences between untreated OVX rats and PTH- or RAL-treated rats after 4 months of treatment still were statistically significant, but the estimates of the differences were reduced to 62% and 76%, 85% and 85%, and 81% and 84% of the initial model estimates. Estimates of the reduction in vertebral maximum load measures after treatment withdrawal did not change with inclusion of these three predictors. Adding vertebral Tb.Th as a predictor did not greatly change any of the estimates (data on file).

## Discussion

Treatment of 6-month-old OVX rats with ALN, hPTH(1–34), and RAL for 4 months increased the compressive strength of the vertebral body and bending load of the tibia. Only animals in the ALN group maintained the vertebral compressive load 4 months after withdrawal of treatment, whereas only PTH treatment maintained the tibial strength. Also, only animals in the ALN group maintained their gain of bone mass up to 8 months after withdrawal of treatment.

A number of animal studies have reported rapid loss of trabecular bone after discontinuation PTH treatment.(10,19,20) The duration of estrogen deficiency, baseline bone turnover, and age of the animals all influence the skeletal response to PTH withdrawal. In this study, the animals were treated with a relatively low dose of PTH (20 µg/kg three times per week or 20 µg per rat per week), comparable with therapeutic doses used to treat osteoporotic men and women (0.30 µg/kg seven times per week or about 140 µg per person per week); it is likely that higher doses would have produced larger increments in bone mass that could be maintained for longer periods. However, the general findings from small animal studies is that the anabolic effects of PTH on trabecular bone sites are lost soon after discontinuation of treatment unless an antiresorptive agent is given.(20) The rapid loss of bone mass after withdrawal of PTH treatment has been attributed to rapid depression of osteoblast activity with a temporary increase in bone resorption that exceeds bone formation.(19) Our histology and biomarker analyses confirmed that osteoclast surface and activity were high with PTH and after its withdrawal and suppressed with ALN and RAL and remained suppressed after withdrawal only in ALN group.

Bone strength is one of the important factors that predict clinical fracture risk.(21) Regression modeling to select the best variables to predict bone strength was limited by the fact that BV/TV and the parameters of bone quality were largely interdependent. The bone-quality parameters such as bone size, thickness, trabecular number, and degree of mineralization, were highly correlated with BV/TV, as observed by the correlation coefficients in this experiment and others.(22–24) Clinical studies also have shown that the changes in trabecular bone volume are associated with a greater gain in bone mass with PTH treatment.(25) Our finite-element analyses of rats' vertebral bodies showed that with the trabecular bone loss after OVX, the load-carrying role of the cortical shell increased significantly, and this was positively correlated with vertebral cross-sectional area, a parameter that was greatly affected by increased bone remodeling of estrogen deficiency and further by antiresorptive and anabolic treatment of bone. The distribution of the load-carrying capacity of the vertebral body is complex and occurs through both the trabecular bone and the cortical shell(18); therefore, the evaluation of overall vertebral strength is challenging because each component may respond differently to both osteoporosis and treatment with bone antiresorptive or anabolic agents. Rockoff and colleagues(26) reported that the cortical shell accounted for approximately 45% to 75% of the vertebral strength and that more force was transmitted via the cortical shell with aging. Recent data using high-resolution µCT-based finite-element models on human vertebra indicate significant variations in load-sharing capacity across vertebrae, with a maximum load fraction carried by trabecular bone occurring near endplates and that of cortical bone at the mid-transverse sections.(18) Although the direction of functional loading is likely to be different in rat and human vertebrae, with the endplates removed during the ex vivo compression of our model vertebrae, the cortical bone may have further contributed to the overall strength, as supported by our finite-element analyses.

All drug treatments improved bone structural and material strength; however, only PTH improved the vertebral yield stress. From a clinical perspective, the skeleton may benefit further from such an influence when it comes to fracture resistance. Higher yield stress means that the elastic properties of bone allow further strain for a given stress prior to failure. In other words, the ultrastructure of the material within mineral and matrix is better able to resume its original shape on removal of the load.(27) This to a large part is a quality of bone from younger individuals, but likely at the expense of taking lower loads,(28,29) whereas with PTH treatment the bone maximum load also was increased. The bone under influence of intermittent PTH has been shown, using quantitative backscattered electron imaging, to have an increased percentage of newly formed matrix of lower mineral density,(30) which may explain the higher yield load observed here and in other preclinical models.(31) Interestingly, our laboratory group has reported previously that daily PTH treatment of OVX rats for 6 months resulted in a more heterogeneous distribution of mineral across the trabecular bone surface compared with OVX rats treated with bisphosphonates or placebo, providing additional support for a new bone with a lower mineral concentration resulting in improvement in yield loads.(32) However, in this study we used a relatively low dose of PTH and a shorter treatment period compared with our previous experiment. We did not observe significant changes in the trabecular BMD following PTH treatment. However, mineral density in cortical bone increased with PTH treatment and not with ALN or RAL treatment, which potentially may explain the superior antifracture efficacy of PTH on cortical bone compared to antiresorptive treatments.

An important clinical consideration in patients with osteoporosis is to maintain bone strength after the bone-active agents are discontinued. After withdrawal of treatments, ALN maintained cancellous, while PTH maintained cortical bone strength up to 4 months. The mechanical improvements with RAL were limited only to the treatment period and disappeared on withdrawal of the treatment. Fuchs and colleagues(9) also treated OVX rats with bisphosphonates for 8 weeks and evaluated the animals 16 weeks after the study medication was withdrawn and found that while there were reductions in lumbar BMD and surface-based bone turnover, the ultimate force of lumbar vertebrae remained higher in the treated animals compared with the controls.

Black and colleagues(4) reported in the Fracture Intervention Trial Long-term Extension (FLEX) that osteopenic women who discontinued alendronate after 5 years showed a moderate decline in hip BMD of 2.4% and in lumbar spine BMD of 3.7% and an increase in the bone resorption marker cross-linked C-telopeptide (CTX-1) of 55.6%. But all BMD and turnover measurements remained at or above pretreatment levels 10 years earlier. In addition, there was no higher fracture risk in the women who discontinued the ALN compared with those who continued ALN, suggesting that there was maintenance of bone strength after the bisphosphonate was discontinued at an anatomic site with a high fraction of trabecular bone even with some reduction in BMD and increase in bone turnover. Our preclinical results support the clinical observation that at least 4 months after ALN was discontinued in rats, bone strength was maintained at the lumbar spine. Additional studies are now required to assess the duration of the preservation of bone strength after the discontinuation of bisphosphonates because this will be a useful guide for clinicians on how to cycle antiresorptive agents and maintain bone strength and fracture risk reduction.

Osteoporotic patients treated with PTH fragments generally have a robust increase in lumbar spine bone mass and reduction in incident vertebral fractures. A number of clinical studies have reported that after PTH treatment is discontinued, there is a reduction in lumbar spine BMD if an antiresorptive agent is not instituted or continued.(6,33,34) However, Lindsay and colleagues(7) reported that postmenopausal women with osteoporosis treated with PTH for a mean of 18 months had a reduction in vertebral fracture risk that persisted for at least 18 months after discontinuation of the therapy despite some loss of lumbar spine BMD with discontinuation of PTH.(35) Our preclinical study did not support these findings.

Interestingly, in our study, 4 months after the PTH treatment was withdrawn, the bending strength of the tibia remained higher than that in untreated animals. While PTH treatment rapidly adds new bone to trabecular and endosteal surfaces, it also induces increased Haversian remodeling with resulting cortical porosity that is dose-dependent.(36–39) After PTH treatment is discontinued, the remodeling space can fill in, and this could result in an increase in cortical bone mass and bone strength.(40,41) In a study by Black and colleagues,(5) treatment of postmenopausal women with osteoporosis for 1 year with PTH fragments followed by 1 year of placebo treatment resulted in no change in total hip and femoral neck BMD measured by DXA and cortical hip volume measured by quantitative CT (QCT), whereas BMD and bone mineral content of the cortical bone of the hip measured by QCT decreased from 1.4% to 3%; this suggested that bone size was maintained after the treatment. Femoral bone strength, estimated by finite-element modeling, was maintained with 1 year PTH treatment followed by 1 year of placebo.(25) We used a low dose of PTH and did not observe a significant amount of elevated Haversian remodeling; however, we did find an increase in cortical bone cross-sectional moment of inertia and thickness with PTH treatment that was maintained after 4 months of withdrawal, which may explain in part the higher tibial bending load.

Our study has a number of strengths, including the evaluation of bone-active agents currently used in clinical practice for the treatment of osteoporosis, a number of sophisticated outcome measures of bone quality, and a study design that evaluated bone strength and quality after 8 months of drug withdrawal. However, there are also a number of shortcomings. First, we used a relatively low dose of hPTH(1–34), making our data difficult to compare with other animal studies. We only evaluated one dose of each bone-active medication, so the effects of dose on aspects of bone quality could not be provided. Also, we had a number of endpoints that required ex vivo samples, so we could not perform repeated measures on these endpoints on the same animals. However, we had a sufficient number of animals in each group to have more than 80% power to test our specific aims. Last, we did not perform any cell-based studies to better understand how these bone-active medications influence cellular mechanisms. Additional studies that incorporate the effects of these medications on bone cell and bone strength measures are needed.

In summary, in our OVX rat model, RAL and PTH treatments were shown to induce changes in trabecular bone that disappeared rapidly after cessation of the treatments, whereas the higher trabecular bone mass following ALN treatment was sustained for 8 months after withdrawal of the treatment. After withdrawal of the treatments, ALN maintained trabecular bone strength, whereas PTH maintained cortical bone strength up to 4 months; the mechanical improvements with RAL were limited only to the treatment period. Additional studies are required to determine the long-term effects of bone strength after discontinuation of osteoporosis medications and to discern how the combination or cyclic therapies will provide better treatment options to improve the long-term treatment of osteoporosis.
